# Personalized labeling: A strategy for supporting self-medicating patients’ decision-making during selection and use

**DOI:** 10.1371/journal.pone.0329725

**Published:** 2026-02-02

**Authors:** Lanqing Liu, Mark W. Becker, Sukhdeep Singh, Dangkamol Wongthanaroj, Laura Bix

**Affiliations:** 1 School of Packaging, Michigan State University, East Lansing, Michigan, United States of America; 2 Department of Psychology, Michigan State University, East Lansing, Michigan, United States of America; 3 Department of Plant, Soil and Microbial Sciences, Michigan State University, East Lansing, Michigan, United States of America; Bangladesh Sericulture Research & Training Institute, BANGLADESH

## Abstract

Interactions between self-medicating consumers and over-the-counter medication (OTC) influence the quality of information processed and, hence, the appropriateness of medication-use decisions. Previous work has shown that information deemed necessary for the safe and effective use of an OTC, information that is required to be present in the Drug Facts Label (DFLs), is often overlooked, and that decision-making related to OTC use could be improved. We proposed the concept of “personalized labeling” to address these shortcomings. This strategy uses an augmented reality interface which presents users with recommendations related to an OTC’s appropriateness for that individual’s use They receive the recommendations via a smart phone app that allows them to point their camera at packages on the store shelf that they are considering purchasing. Specifically, a virtual green check mark or a red stop sign is imposed over the interrogated product after the theoretical app recognizes the OTC and compares product specific warnings with the individual’s health history and medication usage. To develop proof of concept evidence for this strategy, we utilized a computer-based *absolute judgement* task*.* Seventy-two participants were randomly assigned to either a concept-educated group (educated on how personalized labeling would work) or a control group (uninformed about the personalized labeling strategy). Both groups viewed stimulus which included both standard and personalized labels to make binary decisions (yes/ no) related to a drug’s appropriateness for use by a theoretical patient considering single-ingredient OTC products. Decision accuracy and decision time were measured as indicators of labeling effectiveness and efficiency, respectively. Results showed that within the concept-educated group, participants made significantly more accurate (ME = 0.977, SE = 0.007) and faster (ME = 9.584s, SE = 0.854) decisions with the personalized label when compared to the standard label (accuracy: ME = 0.933, SE = 0.017; p = 0.002; time: ME = 19.052s, SE = 2.322; p < 0.001). In contrast, the control group showed no significant difference in accuracy or decision time between the two label types. Additionally, participants across both groups took longer to correctly answer “Yes” (appropriate for use) compared to “No” (not appropriate for use), reflecting that decision time are faster for target-present conditions than target-absent conditions. While educating consumers about the tool seems important, it overlooks that they must install the app and share their health and medication information to use it. As such, we interpret this finding as evidence that personalized labeling could significantly improve consumer decision-making related to the identification of appropriate products. However, future studies are needed with a broader range of populations, package types, use contexts, and real-world conditions.

## Introduction

Beginning in the 1990s, a Drug Facts Label (DFL), a comprehensive, standardized label containing critical information specific to medications sold over the counter, became required on most over-the-counter (OTC) medications sold in the United States. DFLs are intended to provide a systematized, consumer-focused way to display information critical for the safe and effective use of OTC products at the points of purchase and use. Effective labeling is particularly important for OTC medications, as they are deemed to be safe and effective without a healthcare provider’s supervision *when used as directed.*

That said, DFLs have been criticized through the years. Critiques of the existing approach include: the use of small print [1,2] and crowded formatting with extraneous information present [1]; complex wording [2,3] that results in poor comprehension; a tendency of consumers to preferentially attend marketing information [4–7]; and designs that are not optimized for those with poor health literacy [8–10] or who speak English as a second language [11,12]. In short, the DFL needs to be improved.

Recognizing these critiques, Catlin and Brass (2018) conducted an analysis of the existing literature related to DFL design. The authors conclude that even DFL designs that perform “reasonably well” in comprehension studies (required by the US Food and Drug Administration (FDA)) do not communicate effectively in more complex or multifaceted contexts, such as self-selection or actual use studies. This led the authors to conclude that comprehension studies should be viewed as “idealized estimates” of DFL performance. They indicate the “static and non-customizable” nature of current DFLs prevents them from “uniformly meeting the needs” of several populations, including those with limited literacy, the visually impaired, people with language barriers, older consumers, and those with pre-existing believes and attitudes that override DFL messaging [3]. The authors also hypothesize that the multi-attribute nature of information processing complicates decision-making and creates difficulty for consumers who struggle to integrate many factors to arrive at an appropriate, specific decision.

**Fig 1:**
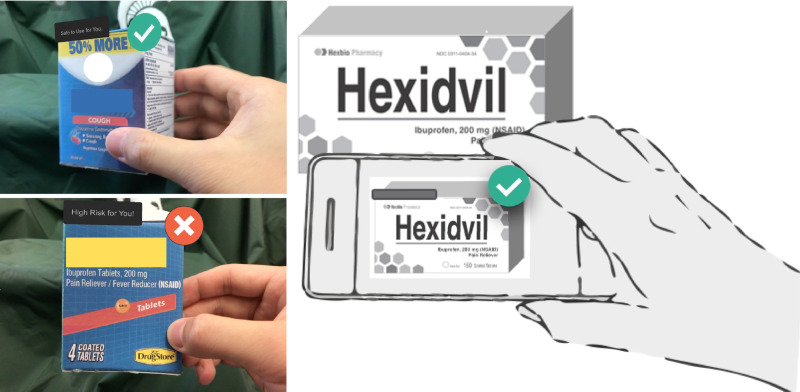
Personalized labeling strategy with the use of augmented reality.

We theorize that the use of a smart device containing an app loaded with the health history and medication use of an individual that employs augmented reality (AR) to return a customized response could help transcend the shortcomings of traditional printed labels, addressing the concerns identified by Catlin and Brass’ (2018). It would not only overcome the shortcomings of the stagnant label design but also better serve the vulnerable audiences highlighted by the authors. Under such a paradigm, a consumer would “interrogate” a potential medication utilizing visual recognition technology in their smart device [3]. The device would recognize the product, cross-reference any contraindications from the label with the user’s health and medication history, and return a customized response (yes = appropriate; no = not appropriate) using AR, which “floats” above the interrogated product. (See Fig 1)

To sum, we envision a label that is flexible, or “personalized”, adapting case-by-case by cross-referencing the drug under consideration to information individual to the person deliberating its use.

Herein, we evaluate the potential benefits that such a strategy could provide in terms of effectiveness (the decision accuracy) and efficiency (decision time to make a correct decision). To obtain these dependent variables, we performed a computer-based, absolute-judgement task in which participants were presented with hypothetical OTC use scenarios and asked to determine whether each medication was appropriate for use by the described individual (answer type: yes/no) when presented with either a standard labels (Front-of-Pack, FOP + DFL) or a personalized label (FOP + DFL + AR indicator).

### Objectives

The overarching objective of this research is to enhance OTC labeling in ways that address problematic issues that are well-documented in the literature. Here, we develop data to support a personalized labeling strategy as a proof of concept. This experiment is framed within the context of ISO/IEC 9241−210, “Ergonomics of human-system interaction- Part 210: Human-centered design for interactive systems.” The ISO standard suggests that usability is a confluence of three factors: *(1) effectiveness,* the “accuracy and completeness with which users achieve specified goals,” *(2) efficiency,* the “resources used in relation to the results achieved,” and *(3) satisfaction*, the “extent to which the user’s physical, cognitive and emotional responses that result from the use of a system, product or service meet the user’s needs and expectations.”

We assess two of the three usability metrics; namely, the effectiveness (decision accuracy) and efficiency (decision time) when interacting with OTC labels. Each dependent variable allows comparison between the standard labek; current use in commercial practice (FOP + DFL), and personalized label (FOP + DFL + Ar indicator); introduced in this study.

## Materials and methods

### 1. Participants and recruitment

Procedures were conducted in accordance with approvals provided by the Michigan State University Office of Regulatory Affairs Human Research Protection Program (STUDY00005057) as exempt. The recruitment period ran from September 12, 2021, to October 1, 2021, and data collection occurred from September 20, 2021, to October 4, 2021. Participants were presented with the IRB-approved consent form and rendered written consent prior to participation. All data were collected anonymously using participant ID numbers, with no personally identifiable information recorded.

Power estimates for this study were based on previous work [13] suggesting an effect size of d = 0.84. The previous work focused on surgical technologists and healthcare providers who were required to select appropriate medical devices for specific scenarios (e.g., latex allergic patient). The power estimate was conducted with 30% of the measured effect size for this study which employed a more general population, or d = 0.25. It was determined that a minimum of 66 participants were required to achieve a power >0.8 at α = 0.05.

Seventy-two participants were recruited via the MSU College of Communication Arts and Sciences SONA recruiting system, distribution of IRB approved flyers, and word of mouth. Participants were eligible for this study if they were: legally sighted, 18 years or older, having used OTC medications within 6 months of the experiment, and having transportation to campus, where the study took place. Participants were characterized using a brief demographic survey followed by tests of their: (1) near-point visual acuity; (2) adult literacy in Medicine (measured with the revised REALM-R); and (3) color differentiation test.

The design was a 2 (Concept-educated/uneducated control) x 2 (Label Strategy: Personalize/standard) design where education was a between subjects factor and label strategy was a within subjects factor. Participants were randomly assigned to the “concept-educated” and a “control” group (n = 36 per group) (see Table 1 for demographics). The concept-educated group was given a brief explanation of the personalized labeling strategy and that the test was meant to explore the usability of an AR-based application and would be operated by the person in the scenario with answers returned on their smart device. They were informed about the meaning of two visual indicators used: a green checkmark, indicating the product is appropriate for use, and a red stop sign, indicating the product is not appropriate for use. Participants in the control group were not provided any information about the concept or the use of symbols. For both groups, all trials displayed a standard label (the typical FOP alongside the standard DFL), with half of the labels augmented by personalization (the floating red stop sign or green checkmark).

**Table 1:** Frequency table- participants across demographic factors by group assignment.

Characteristic	Group Assignment	Value	Number	% of Total (72)
**Sample size**	**Concept-educated**	N1	36	50%
	**Control**	N0	36	50%
**Sex**	**Concept-educated**	Male	8	11.1%
		Female	28	38.9%
	**Control**	Male	11	15.3%
		Female	25	34.7%
**Color Differentiation**	**Concept-educated**	Normal	36	50.0%
		Color blind	0	0.0%
	**Control**	Normal	35	48.6%
		Color blind	1	1.4%
**Education**	**Concept-educated**	Master’s or higher	14	19.4%
		Bachelor or lower	22	30.6%
	**Control**	Master’s or higher	8	11.1%
		Bachelor or lower	28	38.9%
**Ethnicity**	**Concept-educated**	White	22	30.6%
		Others	14	19.4%
	**Control**	White	24	33.3%
		Others	12	16.7%
**Health Literacy**	**Concept-educated**	Normal	35	48.6%
		Risk for poor literacy	1	1.4%
	**Control**	Normal	35	48.6%
		Risk for poor literacy	1	1.4%
**Native Language**	**Concept-educated**	English	27	37.5%
		Others	9	12.5%
	**Control**	English	30	41.7%
		Others	6	8.3%
**Visual Acuity**	**Concept-educated**	Normal (≤ 20/40)	36	50.0%
		Poor (>20/40)	0	0.0%
	**Control**	Normal (≤ 20/40)	36	50.0%
		Poor (>20/40)	0	0.0%
**Age**	**Concept-educated**	Mean (Min, Max)	34.64 (19, 65)	
		Std. Deviation	13.226	
	**Control**	Mean (Min, Max)	34.19 (20, 73)	
		Std. Deviation	14.903	

### 2. Stimuli and trials

**Fig 2:**
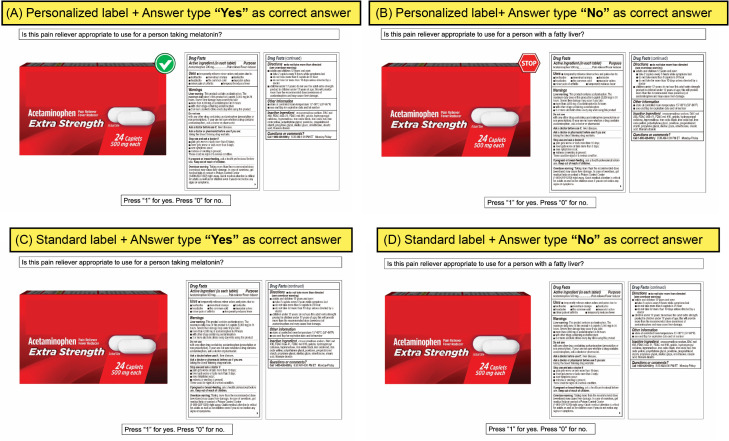
Example of a single-ingredient acetaminophen product shown in four treatments. (A) Personalized label with “Yes, appropriate for use”. (B) Personalized label with “No, not appropriate for use”. (C) Standard label with “Yes, appropriate for use”. (D) Standard label with “No, not appropriate for use”.

A custom-built program (E Prime 3.0 Tools Psychology Software; Pennsylvania USA) was used to create an absolute judgment task that recorded both correct and incorrect answers as well as decision time. Eight single-ingredient OTC products were utilized as the basis for the test trials as they represent some of the most commonly purchased and self-medicated OTCs across major therapeutic categories: acetaminophen (ACE—scientifically referred to as APAP) – Tylenol; ibuprofen (IBU) – Advil; naproxen (NAP) – Aleve; guaifenesin (GUA) – Mucinex, omeprazole (OME) – Prilosec; phenylephrine (PHN- scientifically referred to as PE) – Sudafed; cimetidine (CIM) – Tagamet; ranitidine (RAN) – Zantac. Each product was depicted a total of four times; once where the answer type was “Yes, appropriate”, and once where the answer type was “No, not appropriate” in the standard label, and these same conditions were repeated in the personalized label. As such, each participant (regardless of whether they were in the control group or the concept educated group) evaluated 4 trials per active ingredient (See Fig 2 for a single set affiliated with one product), resulting a total of 32 test trials (8 active ingredients × 2 label designs × 2 answer types).

**Fig 3:**
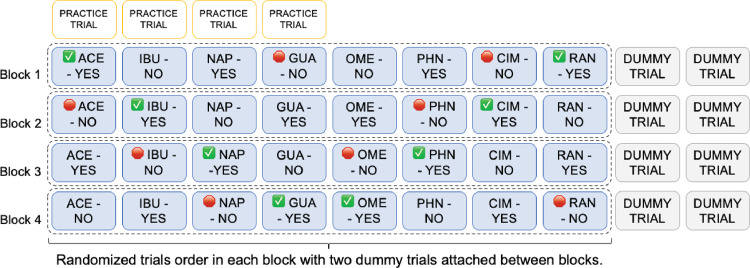
Stratified randomization scheme of the absolute judgement task.

To minimize order effects, a stratified-randomization pattern was used (See Fig 3). The four treatments of each product were distributed so each appeared once in one of four experimental blocks. Each block contained one version of every active ingredient, ensuring that the same active ingredient was never presented back-to-back in consecutive trials. Within each block, trial order was randomized. Dummy trials, involving questions about the supplement products, were inserted between experimental blocks to prevent repeated exposure to the same active ingredient that appeared at the end of one block and at the beginning of the following block. Practice trials were also added prior to the first experimental blocks to allow participants to become familiar with the trial format. These practice and dummy trials were not included in the data analysis, as they simply provided a practice before test trials and a break between test trials so that the same drug could not appear in consecutive trials at any point througout the study. This approach reduced the likelihood of short-term memory effects that could influence participant responses.

Participants were instructed to assume that the person in the scenario had the condition/symptoms that the drug treats and answer whether the presented medication was appropriate for that person to take by pressing a “1” for “yes, appropriate” and “Z” for “No, not appropriate” on an external keypad attached to the test computer.

### 3. Statistical analysis

A generalized, linear mixed model (GLMM) was fitted to assess the influence of the variables of interest on two outcomes (1) answer accuracy, the probability of correctly identifying whether a product was appropriate for the given scenario, and (2) decision time, the duration required to arrive at the correct answer. For answer accuracy, binary data (correct/ incorrect) were interpreted in terms of probability (p) of correctly answering trial questions with logit transformation $ln\left(\frac{p}{\text{1}-p}\right)$ to meet probability assumptions. Estimated means were back-transformed and reported in the original target scale.

The decision time data were checked for the validity of normality assumptions prior to statistical analyses. Residual plots and normal probability plots of the original data suggested an appropriate transformation, requiring a natural log transformation. Tukey’s method was used for minor non-constant variance, and Satterthwaite’s method was used to adjust degrees of freedom. A GLMM was then fitted to this natural log-transformed decision time variable.

The predictor variables included in both of the final models were: group (concept-educated/control), label design (personalized/standard), answer type (yes/no as a correct response), active ingredients, and demoraphic covariate (education, ethnicity, sex, language, and age). All possible 2-way, 3-way interactions among group, label design and answer type were also included in each model. Additionally, the following covariates were included in the final model, with participants treated as random effects. All estimated means were back-transformed to the original scale of the dependent variable; in percentage for answer accuracy, and seconds for decision time.

## Results

### 1. Participant characterization

Table 1 provides participant frequencies across demographic factors by group type for factors of interest across the entire study population.

### 2. Answer accuracy

A total of 2,304 trials (72 participants × 32 trials) were analyzed in this absolute judgement task to examine data for effects on answer accuracy, defined as the probability of correctly identifying the correct answer in given scenario (correctly identifying as appropriate or correctly identifying as inappropriate). Across all trials, participants responded correctly in 2,141 trials (92.9%) and incorrectly in 163 trials (7.1%).

A summation of the statistical analysis is presented in Table 2. Significant main effects were identified for label design (p = 0.002) and active ingredient (p = 0.000) on answer accuracy. Not surprisingly, a significant interaction between group and label design was identified (p = 0.003). No other main or interaction effects rose to the level of significant.

**Table 2:** Tests of fixed effects on answer accuracy.

*Effects*	*F*	*df1*	*df2*	*Sig.*
*Corrected Model*	3.036	19	2284	0.000
*Group*	1.227	1	2284	0.268
***Label Design***	**9.553**	**1**	2284	**0.002**
*Answer type*	0.526	1	2284	0.468
***Active Ingredient***	**4.985**	**7**	2284	**0.000**
*Education*	0.243	1	2284	0.622
*Ethnicity*	0.433	1	2284	0.510
*Sex*	0.222	1	2284	0.638
*Language*	2.174	1	2284	0.140
*Age*	0.012	1	2284	0.912
Group * Label Design	**8.567**	**1**	2284	**0.003**
Group * Answer type	0.820	1	2284	0.365
Label Design* Answer type	0.018	1	2284	0.893
Group * Label Design * Answer type	0.089	1	2284	0.765

**Bolded effects** are significant at 0.05. Probability distribution: Binomial; Link function: Logit.

**Fig 4:**
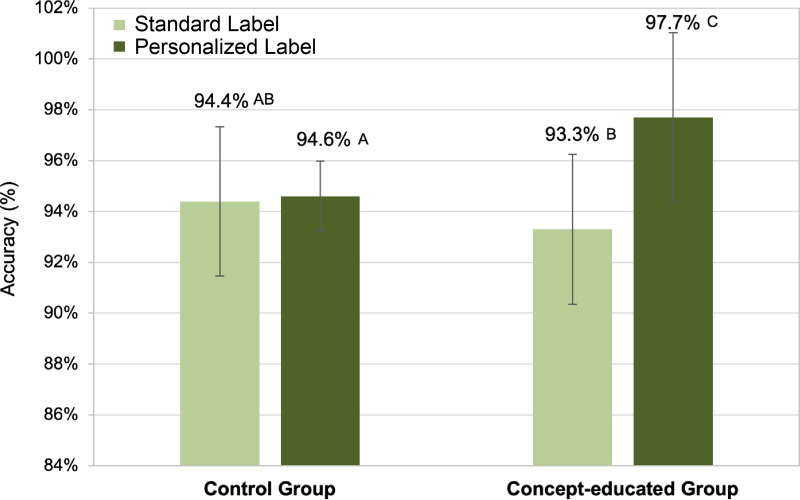
Estimated means of answer accuracy (measure of effectiveness) by group and label design. Error bars represent the standard error and the variables with differing letters indicate statistical significance at α = 0.05.

Pairwise comparisons were conducted to interpret the significant 2-way interaction between group (concept-educated vs. control) and label design (personalized vs. standard) (See Fig 4). When the personalized label was introduced prior to the test (i.e., the concept-educated group), answer accuracy was significantly higher when evaluating personalized labels (ME = 0.977, SE = 0.007) compared to the standard labels (ME = 0.933, SE = 0.017) (p = 0.002). In contrast, participants in the control group (uninformed about the personalized labeling strategy) showed no significant difference (p = 0.898) in answer accuracy when their responses for trials employing personalized label (ME = 0.946, SE = 0.015) were compared to the trial responses from the standard labels (ME = 0.944, SE = 0.015). Further, the answer accuracy of trials conducted by the concept-educated group when viewing personalized label was significantly greater compared to all other combinations of group and label design at α = 0.05 (See Fig 4).

Within the covariates explored, the active ingredient was found to significantly affect answer accuracy (p < 0.001). As shown in Tables 3 and 4, participants were least accurate in trials involving acetaminophen (ME = 0.909, SE = 0.023). Pairwise comparisons made to explore the effect further suggested that, when using acetaminophen as reference collapsing across both groups and label designs, accuracy results were significantly lower than trials involving guaifenesin (ME = 0.995, SE = 0.023; p = 0.001), and naproxen (ME = 0.973, SE = 0.010; p = 0.018). Considering the widespread use of acetaminophen in in OTC medications and its potential risks, such as kidney failure from overdose [8], further study is needed to examine how active ingredient and patient characteristics, such as the prior experience, frequency of use or drug familiarity, influence information processing and decision-making.

**Table 3:** Results of estimated means answer accuracy across active ingredients tested.

*Ingredient*	*Mean*	*Std. Error*	*95% Confidence Interval*	
			*Lower*	*Upper*
*Ranitidine (acid reducer)*	0.909	0.023	0.854	0.944
*Phenylephrine (nasal decongestant)*	0.958	0.013	0.922	0.977
*Omeprazole (acid reducer)*	0.948	0.015	0.909	0.971
***Naproxen (pain reliever)***	**0.973**	**0.01**	**0.944**	**0.987**
*Ibuprofen (pain reliever)*	0.916	0.021	0.863	0.949
***Guaifenesin (cough suppressant)***	**0.995**	**0.004**	**0.978**	**0.999**
*Cimetidine (acid reducer)*	0.909	0.023	0.854	0.944
*Acetaminophen (pain reliever)*	0.909	0.023	0.854	0.944

Continuous predictors are fixed at the following values: Age = 34.42.

**Table 4:** Simple contrasts of answer accuracy comparing each active ingredient to acetaminophen.

*Ingredient*	*Contrast Estimate*	*Std. Error*	*t*	*df*	*Adj. Sig.*	*95% Confidence Interval*	
						*Lower*	*Upper*
*Ranitidine (acid reducer)*	−1.33E-15	0.024	−5.57E-14	2284	1	−0.047	0.047
*Phenylephrine(nasal decongestant)*	0.049	0.022	2.266	2284	0.118	−0.007	0.105
*Omeprazole (acid reducer)*	0.04	0.022	1.817	2284	0.278	−0.015	0.094
***Naproxen (pain reliever)***	**0.064**	**0.022**	**2.967**	**2284**	**0.018**	**0.007**	**0.121**
*Ibuprofen (pain reliever)*	0.007	0.023	0.292	2284	1	−0.049	0.063
***Guaifenesin (cough suppressant)***	**0.086**	**0.022**	**3.876**	**2284**	**0.001**	**0.026**	**0.146**
*Cimetidine (acid reducer)*	−1.33E-15	0.024	−5.57E-14	2284	1	−0.047	0.047

The sequential Bonferroni adjusted significance level is 0.05. **Bolded text** represents significance at α = 0.05. Confidence interval bounds are approximate.

### 3. Decision time

While accuracy represents a measure of the label design’s effectiveness, the decision time (time required to reach correct answer) measures the efficiency [14]. Only 2,141 correctly answered test trials were included in the analysis that considered time to correct decision as the independent variable (i.e., only those that involved a correct response were included).

Results for decision time are presented in Table 5. Significant fixed effects were noted for group (p < 0.001), label design (personalized vs standard) (p < 0.001), answer type (appropriate for use under a given scenario, yes/no) (p < 0.001), and active ingredients (p < 0.001). Additionally, *all 2-way interactions* significantly impacted the time to correct decision, namely, group × answer type (p = 0.009), group × label design (p < 0.001), and answer type × label design (p < 0.001). The 3-way interaction was also found statistically significant; group × answer type × label design (p = 0.015).

**Table 5:** Tests of model effects on time to correct decision responses.

*Source*	*F*	*df1*	*df2*	*Sig.*
*Corrected Model*	29.035	19	204	0.000
***Group***	**21.629**	**1**	**65**	**0.000**
***Label Design***	**111.276**	**1**	**2056**	**0.000**
***Answer Type***	**172.187**	**1**	**2057**	**0.000**
***Ingredient***	**15.819**	**7**	**2057**	**0.000**
*Education*	3.8	1	65	0.056
*Ethnicity*	0.811	1	65	0.371
*Language*	2.711	1	65	0.105
*Sex*	0.13	1	65	0.719
*Age*	0.263	1	65	0.610
Group * Answer Type	**6.769**	**1**	**2057**	**0.009**
Group * Label Design	**107.43**	**1**	**2056**	**0.000**
Answer type *Label Design	**12.398**	**1**	**2057**	**0.000**
Group * Answer type * Label Design	**5.928**	**1**	**2057**	**0.015**

**Bolded text** represents significance at α=0.05.

**Fig 5:**
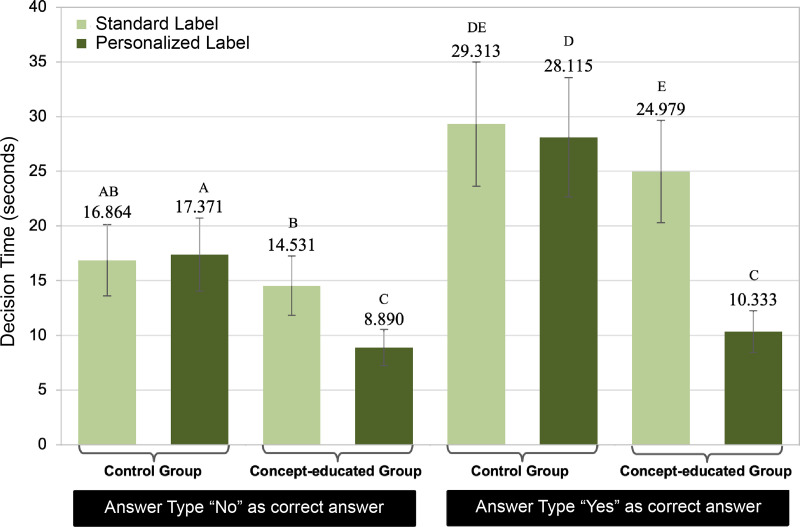
Estimated means of decision time on correct decision-making across group, answer type, and label design. Error bars representing the standard error and the variables with differing letters indicate statistical significance at α = 0.05.

To interpret the 3-way interaction that resulted in a significant effect on time to correct decision response; group (concept-educated vs control) × label design (personalized vs standard) × answer type (appropriate for use under a given scenario, yes/no), pairwise comparisons were analyzed. Fig 5 provides a visual assessment of this interaction.

For all comparisons of answer type (appropriate yes/no) across the other variables: the concept-educated group × personalized label; the concept-educated group × standard label; control group × personalized label; control group × standard label), participants took significantly longer to correctly respond to the affirmative trials (“Yes, appropriate”) compared to the negative trials (“No, not appropriate”) as the correct answer. This is consistent with research that investigates target absent vs target present responses [15–18] (i.e., products eliciting a yes/appropriate response are target absent; that is, there is no warning or contraindication information to find. As such, the “yes” takes significantly longer than the “no” where there is a warning against using the product.

Data also supported the finding that participants in the concept-educated group took significantly less time to make decisions than those in the control group, regardless of whether the correct answer was affirmative, “Yes” (appropriate for the scenario) or negative, “No” (not appropriate for the given scenario). When collapsing the data across the label design (personalized and standard), participants in the concept-educated group made decisions faster for both answer types. For affirmative responses, the average decision time of concept-educated group (p < 0.001; ME = 17.656, SE = 1.825) was significantly shorter than those in the control group (ME = 28.714, SE = 3.002. Similarly, for negative responses, the concept-educated group yielded a faster average decision time (p < 0.01; ME = 11.711, SE = 1.260) compared to the control group (ME = 17.118, SE = 1.791). These findings reflect that the concept-educated group had shorter decision times, regardless of the answer type or the label design. Details of the pairwise comparisons for this analysis are shown in Fig 5.

The faster responses were yielded in the personalized labeling strategy that occurred when the affirmative responses (“Yes”) were gathered within the concept-educated group. In this condition, participants made decisions significantly faster (ME = 10.333, SE = 0.979), than participants in the control group (ME = 28.115, SE = 2.778), an average difference of 17.782 seconds. This demonstrates that the highest efficiency gain was achieved when both the concept education and the personalized labeling were associated.

To further explore the 3-way interaction, we also compared the effect of personalized label and the standard label with each group × answer type combination. Within the control group, results did not indicate a significant difference (p = 0.530) in time to correct decision when trials with personalized labels (ME = 28.115, SE = 2.778) were compared with trial times from the standard labels (ME = 29.313, SE = 2.895). This was not the case for the concept-educated group, where a significant difference was observed. When the correct answer was “yes”, participants spent 14.646 seconds less on average (p = 6.66E-15) when the trials used personalized label (ME = 10.333, SE = 0.979) compared to a standard label (ME = 24.979, SE = 2.388). Similarly, when the correct answer was “no”, participants made decisions 5.641 seconds faster on average (p = 1.53E-09) when trials used a personalized label (ME = 8.890, SE = 0.842) compared to the standard label (ME = 14.531, SE = 1.387).

Results suggest that participants responded faster on a target-present searches. That is, searches where they had to find a specific target representing a problem for the person in the scenario (answer “no” do not take this) than on a target-absent search; as compared to target absent searches (correct answer yes, ok to take). This pattern aligns with published, visual search literature; where decision times are faster for target-present conditions than target-absent conditions [15].

## Discussion

We are in the era of the metaverse, a time when new technologies allow and enable us to connect and experience things in ways never imagined, impacting how we solve problems and make decisions. At the same time, dramatic changes are occurring in our approach to healthcare. The age of “personalized medicine” leverages the paradigm that the individualized and nuanced characteristics of a patient require interventions that are tailored to a patient’s specific molecular and physiological characteristics in order to yield optimized outcomes [19].

Within this context, we consider the state of OTC labeling. Extraneous information presented in the “one size fits all” approach to the DFL potentially interferes with a given patient’s ability to find information critical for *their unique condition and circumstance*. Catlin and Brass’ (2018) review of OTCs and their observation that “the DFL by nature is static, non-customizable, text-based communication tool,” that does not “uniformly meet the needs of specific populations” (e.g., limited literacy, limited vision, older consumers or those with language barriers or preconceived notions related to the drug), suggests the need for a new archetype for labeling [3]. Trends in emerging technology and increasing customization provide tremendous opportunity to rethink labeling practice to address identified shortcomings and challenges of the current DFL.

Our research goal was to provide objective evidence relating to the benefits of a theorized, personalized labeling strategy. Results suggest that this approach would enhance the effectiveness of decision making (accuracy – See Fig 4) as well as efficiency (decision time – See Fig 5) over the current approach, *when participants are aware of the new paradigm*. That said, the very nature of the application (a theorized app) would mean that patients that utilized it would be aware of it, and, as such, would not need to be educated related to the new paradigm.

The significant three-way interaction of group × label design × answer type catalyzed the need to explore the mediating effect of answer type (whether an affirmative or negative answer was correct) on decision time. Analysis indicated that participants took less time to find answers when “no” was the correct answer compared to those trials where “yes” was correct (See Fig 5). This finding is intuitive, as the questions were designed such that those with “no/not appropriate” as correct answers directly corresponded to key words (warnings and contraindications) in the DFLs; in other words, there are visual targets present for the participant to find. This corroborates the work of others, who indicate that searches take longer when search targets are absent compared to when targets are present in a visual search field [15–18].

Using Tylenol, for example, we asked, “Is this pain reliever appropriate to use for a person with a fatty liver?” In this case, the keyword “fatty liver” corresponded directly to the “liver warning” and the statement “ask a doctor before use if liver disease” on the DFL. Conversely, questions with an affirmative response “yes/ appropriate for use”, would not contain directly correlated information in the DFL; the visual target is absent. For the Tylenol example again, we asked, “Is this pain reliever appropriate to use for a person taking melatonin?” In this case, the key words “taking melatonin” cannot be directly identified in the warning label. This finding is supported by the literature, which suggests that searching for an absent target; no warning present suggesting not to take this product, generally results in longer search times than target present searches [15–18]. It should be noted, however, that the visual target is only absent in the case of the standard labeling as the personalized strategy provides a target (symbol) in the form of a customized response tailored to the individual using the theoretical app.

It is somewhat surprising that participants in the control group did not benefit from the personalized label – one might have expected that they would have realized that a green checkmark were appropriate, while those with a red stop sign were inappropriate. However, our results did not reflect this to be the case. This suggests that, without conceptual framing, users may not automatically assign meaning to color or symbol cues. In reality, individuals using the app that would be required to trigger the personalized response from the product would have some understanding of its purpose, thus, building awareness of the system would not pose a real-world challenge. Moreover, because the labeling system would operate within the application interface, users’ initial setup would naturally provide the conceptual framework informed user to interpret these symbols effectively. From an experimental perspective, the fact that the personalized condition provided a benefit only in those educated about the personalization scheme provides confidence that it is the use of the scheme that led to benefits among the educated group.

The final finding was more unexpected. Namely, the active ingredient had significant impacts on both effectiveness (accuracy) and efficiency (decision time). We hypothesize that this could be mediated by participants’ familiarity with the drug, which could lead to an overconfidence in decision-making rather than deliberate the available information search. Notably, in other studies conducted by our research team, participants were more likely to make problematic decisions related to an OTC product’s appropriateness for their own use when they reported the active ingredient to be familiar to them [20].

## Limitations

It is worth noting several limitations of the experiment. The sample size and participant diversity were limited relative to the broader population of the self-medicating consumers, whose ages, health literacy, and cultural background vary more substantially. Further, while our study afforded an experimental validity in that we could precisely control and balance, yes and no responses, measure time, etc., it comes at a cost of ecological validity. The computer-based task nature of the task did not require participants to make decisions for themselves, nor did they have to load health information of an app or utilize tools like augment reality which may be unfamiliar. It did not require them to utilize the smart device application, but instead was a theoretical assessment of the potential benefit such a program might provide.

It is recommended that future work using the same labeling paradigm be conducted in a more realisitic setting asking consumers to trial the products based on their own health history in a context where OTC product selection occurs. Such a study also has the potential to yield insights into whether (or not) consumers are cavalier in their decision making related to OTC products as some research suggests that consumers view decisions about OTCs as low stakes/risk and do not devote significant time or credence to warning labels on the same.

Finally, while the current study focused on proof of concept, the implementation of the AR-based labeling system in real-world scenario would require consideration of additional factors, such as technological feasibility, user adoption, data privacy, and regulatory compliance.

## Supporting information

S1 File Flat file change detection. (XLSX)

S2 File Flat file absolute judgement. (XLSX)
